# Supplementary material for: Molecular Profiles of Serum-Derived Extracellular Vesicles in High-Grade Serous Ovarian Cancer
Source: Cancers (Basel). 2022 Jul 23;14(15):3589. doi: 10.3390/cancers14153589 (PMC9330879; doi:10.3390/cancers14153589)
Supplement: Supplementary file 1 [file cancers-14-03589-s001.zip › cancers-1829458-supplementary.pdf]

Molecular profiles of serum-derived extracellular vesicles in high-grade serous ovarian cancer

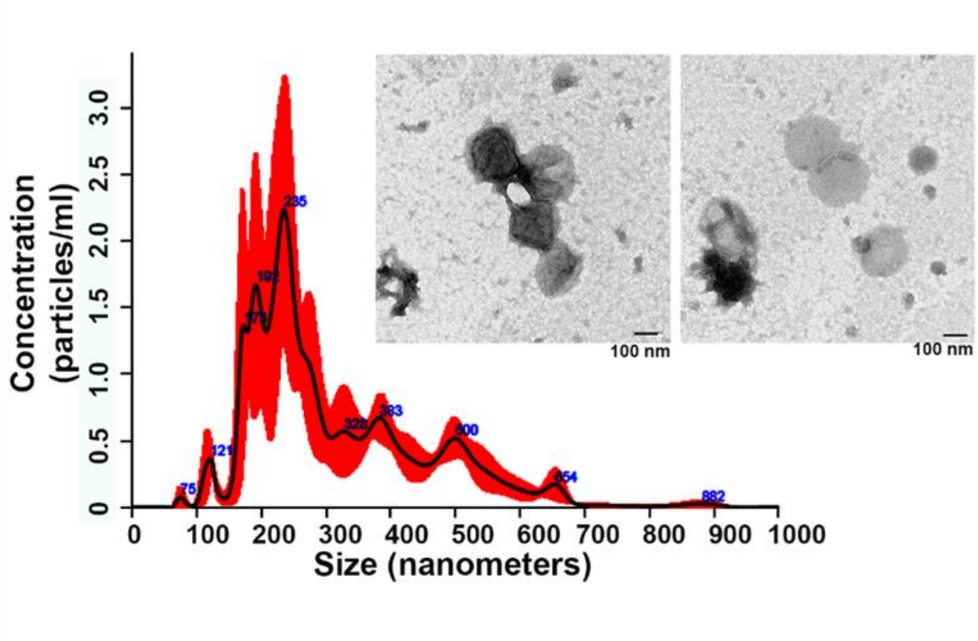

Figure S1: **Nanotracking particle analysis and transmission electron microscopy:** Characterization of m/lEVs by transmission electron microscopy and nanoparticle tracking analysis. Circulating EVs were isolated from patients-derived plasma.

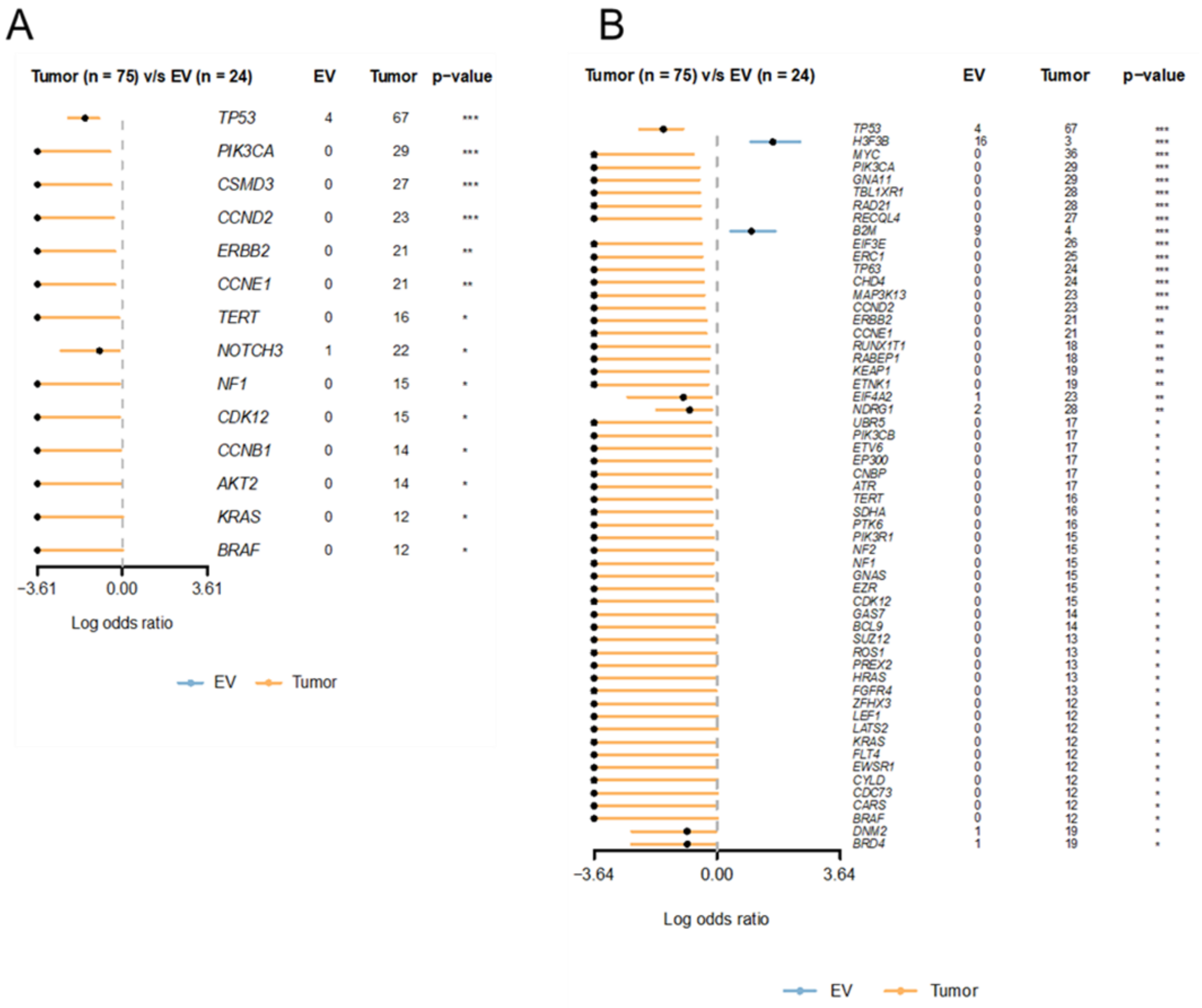

Figure S2. **Somatic alteration comparison between EV-DNA and tumor DNA.** (A) Comparison of the frequencies of somatic alterations in ovarian cancer genes between EV-DNA and tumor DNA. (B) Comparison of the frequencies of somatic alterations in cancer-hallmark genes between EV-DNA and tumor DNA. *= p value <0.05; **= p value<0.01; ***= p value<0.001. Log odds ratio < 0 means the alterations are enriched in tumor and log odds ratio > 0 means the alterations are enriched in EV.

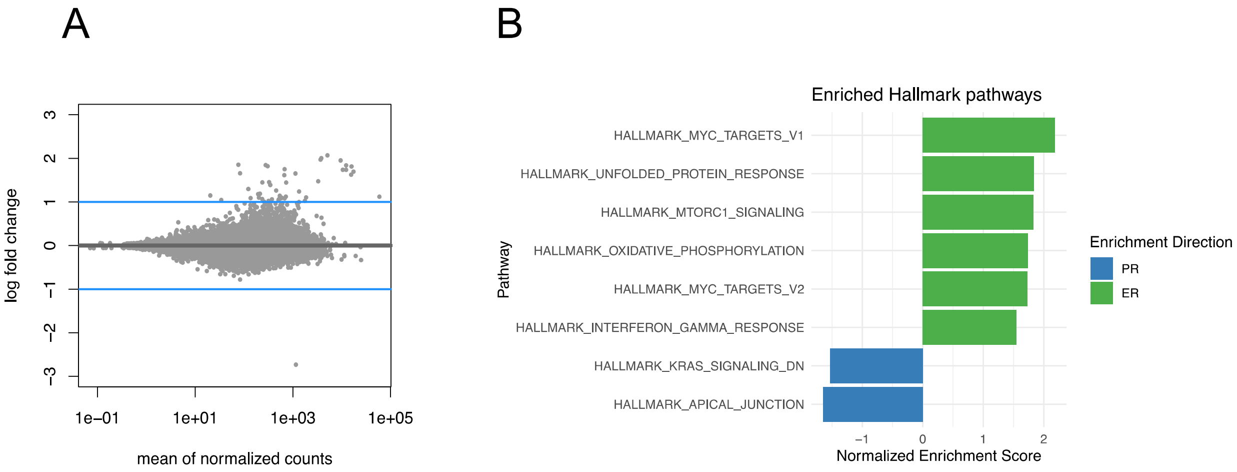

Figure S3. Differential expression analysis in the NACT-ER vs NACT-PR groups. (A) MA plot showing the identified genes, with the blue lines representing the cutoffs for differentially expressed genes (absolute value of L2FC ≥ 1) between the two groups. The blue dots represent the genes with adjusted p-values (adj-p) < 0.05. (B) GSEA analysis shows the enriched cancer-hallmark pathways in the NACT-ER vs NACT-PR groups.

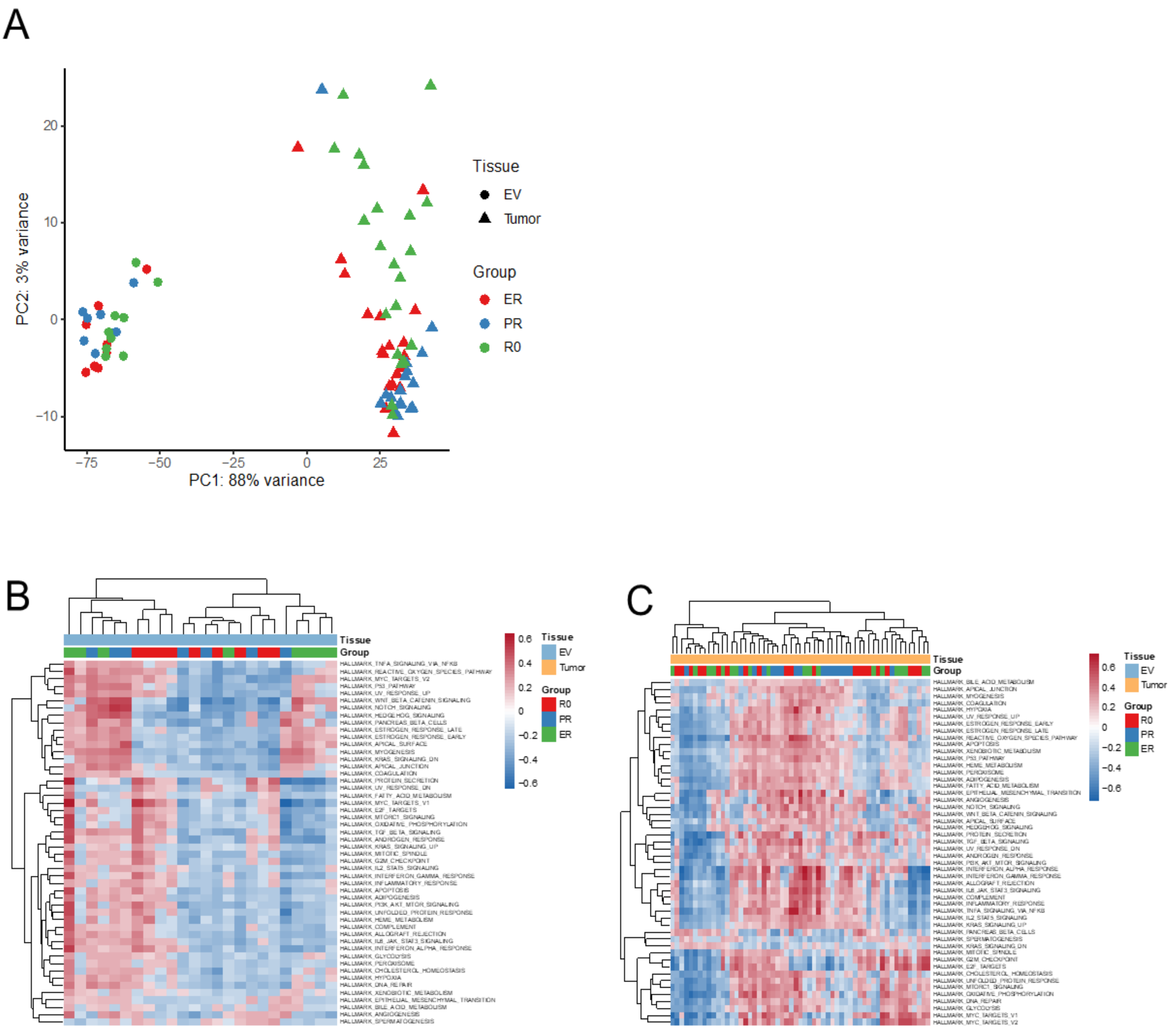

Figure S4. RNA expression profile comparison between EV-RNA and tumor RNA. (A) PCA plot of the transcriptomic profiles of EV-RNA and tumor RNA. EV samples (dots) and tumor samples (triangles) are indicated by patient group. (B) Heatmap of the enrichment of cancer-hallmark pathways in EV-RNA samples. (C) Heatmap of the enrichment of cancer-hallmark pathways in tumor RNA samples.

Table S1: Clinico-pathological characteristics of the cohort. One-way ANOVA was not significant for differences in age, BMI and CA125 (Brown-Forsythe test not significant for age, BMI and CA125, Bartletts's test significant for CA125, p 0.03).

Group	Patient ID	Age (yr)	BMI (kg/m²)	Race	CA-125 at diagnosis (units/ml)	Disease site	Stage	BRCA status
NACT-ER	NACT-ER-1	49	28.9	Asian	26.6	Ovarian	Stage IIIC	Patient declined
	NACT-ER-2	53	29.8	White	1934	Ovarian	Stage IVA	No mutation
	NACT-ER-3	77	22.6	White	465.5	Ovarian	Stage IIIC	No mutation
	NACT-ER-5	59	27.7	White	367.7	Ovarian	Stage IIIC	No mutation
	NACT-ER-6	67	38.4	Black	595.9	Ovarian	Stage IVB	Unknown
	NACT-ER-7	71	21.4	White	740.1	Ovarian	Stage IIIC	No mutation
	NACT-ER-8	78	23.9	White	365.3	Ovarian	Stage IIIC	No mutation
	NACT-ER-9	58	30.4	White	87.6	Ovarian	Stage IIIC	Unknown
	Average	64.0	27.9		572.8			
NACT-PR	NACT-PR-1	62	28.2	White	1116	Ovarian	Stage IIIC	No mutation
	NACT-PR-3	73	18.9	White	335.3	Ovarian	Stage IVB	No mutation
	NACT-PR-4	55	22.8	White	148.6	Ovarian	Stage IIIC	No mutation
	NACT-PR-5	62	29.4	His-panic	1467	Ovarian	Stage IIIC	Patient declined
	NACT-PR-6	73	30.8	White	335.6	Ovarian	Stage IIIC	No mutation
	NACT-PR-7	67	27.7	His-panic	3452	Ovarian	Stage IVA	BRCA2
	NACT-PR-10	59	19.9	White	551.3	Ovarian	Stage IVA	Unknown
	Average	64.4	25.4		1058.0			
R0	R0-1	56	26	White	356.2	Ovarian	Stage IIIC	BRCA2
	R0-3	72	28.9	White	52.1	Ovarian	Stage IIIC	No mutation
	R0-4	38	43.8	White	30.8	Ovarian	Stage IIIC	No mutation
	R0-5	82	23.3	Black	95.5	Peritoneum	Stage IIIC	Unknown
	R0-6	73	32.9	White	122.5	Ovarian	Stage IIIC	No mutation
	R0-7	70	24.8	White	343	Ovarian	Stage IIIC	No mutation
	R0-8	72	31.1	White	1256	Ovarian	Stage IIIC	No mutation
	R0-9	56	18.9	White	908.9	Fallopian tube	Stage IIIA1	BRCA1
	R0-10	60	24.6	White	451.2	Ovarian	Stage IIIC	No mutation
	Average	64.3	28.3		401.8			
